# Correct understanding of the definition and management strategies for refractory hydrocephalus

**DOI:** 10.1186/s41016-025-00403-9

**Published:** 2025-08-18

**Authors:** Zhixiong Lin, Hua Feng, Wangming Zhang, Gelei Xiao, Jingyu Chen, Zhiqiang Liu

**Affiliations:** 1 Department of Neurosurgery, Sanbo Brain Hospital, Capital Medical University, Beijing, Beijing, 100093 China; 2 Department of Neurosurgery, Southwest Hospital, Third Military Medical University, Chongqing, China; 3 The National Key Clinical Specialty, Guangdong Provincial Key Laboratory on Brain Function Repair and Regeneration, Neurosurgery Center, The Engineering Technology Research Center of Education Ministry of China on Diagnosis and Treatment of Cerebrovascular Disease, Zhujiang Hospital, The Neurosurgery Institute of Guangdong Province, Southern Medical University, Guangzhou, China; 4 Department of Neurosurgery, Xiangya Hospital, Central South University, Changsha, Changsha, China

**Keywords:** Hydrocephalus, Diagnosis, Treatment, Refractory

## Abstract

Hydrocephalus refers to the abnormal accumulation of cerebrospinal fluid (CSF) in the central nervous system, typically resulting from an imbalance between CSF production and absorption. Traditional classifications of hydrocephalus do not incorporate management strategies (not classified according to the degree of difficulty of treatment). Clinically, hydrocephalus that is challenging to treat is often categorized as refractory hydrocephalus (RH). However, the absence of a unified definition of RH impedes the standardization of treatment approaches, raising clinical dilemmas. This article explores the definition, etiologies, classification, and management strategies for RH. Based on the literature and the Diagnosis-Related Group payment system principles, RH is clinically defined as progressive hydrocephalus meeting one or more of the following criteria: (1) the absence of significant clinical or radiological improvement within 60 days despite standard interventions, usually due to pathological factors, such as abnormal CSF characteristics, (2) inability to achieve curative surgical treatments attributable to complex anatomy such as abnormal dynamic changes or multiloculated compartments, and (3) failure to respond after two consecutive therapeutic procedures. RH consists of six distinct subtypes, with infectious hydrocephalus being the most common, followed by low-pressure hydrocephalus. Temporary management strategies for RH must be carefully tailored to patient-specific characteristics, considering the risk–benefit analysis of available measures. In cases of infectious RH, achieving CSF sterilization and evaluating the results are crucial. Curative surgery for infectious RH should be performed only after CSF has been completely sterilized to normal levels. In low-pressure RH, a critical focus is identifying and addressing the sites receiving CSF.

## Background

Hydrocephalus refers to the abnormal accumulation of cerebrospinal fluid (CSF) in the central nervous system, often caused by an imbalance between CSF production and absorption [1]. It is broadly classified into primary and secondary types, with secondary hydrocephalus arising from conditions such as intracranial hemorrhage, infection, trauma, or brain tumors. These conditions disrupt normal CSF flow and absorption, leading to ventricular enlargement [2, 3]. Although refractory hydrocephalus (RH) is commonly encountered, it is often regarded by some physicians as a straightforward condition with simple treatment methods such as shunting or ventriculostomy. However, despite the apparent simplicity of these procedures, complication rates for hydrocephalus treatment within the first year range from 20 to 40%, with long-term rates reaching up to 60% [4–7][. Once diagnosed and treated surgically, hydrocephalus patients often require lifelong medical care. Some types of hydrocephalus present exceptional clinical challenges, resulting in prolonged treatment durations, high costs, poor outcomes, and significant burdens on patients and families. Such cases are categorized as RH. Iatrogenic hydrocephalus, in particular, is associated with frequent disputes and Diagnosis-Related Group overpayment issues. 

However, the absence of a unified definition of RH impedes the standardization of treatment approaches, raising clinical dilemmas. Effective management of RH requires meticulous attention. Enhancing the diagnostic and therapeutic strategies for RH is essential to improve the overall treatment of hydrocephalus. Moreover, under the Diagnosis-Related Group/Diagnosis Intervention Packet payment framework, clear definitions, classifications, and treatment principles for RH are crucial.

## Traditional classification of hydrocephalus and definition of RH

The term “hydrocephalus” originates from the Greek words “hydro-” (water) and “kephalos” (head). Hydrocephalus is traditionally classified based on etiology, disease mechanisms, rate of development, intracranial pressure (ICP) changes, compensatory brain functions, pathophysiology, and location [7–10].

- By etiology: Congenital and acquired hydrocephalus.
- By mechanism: Obstructive and communicating hydrocephalus.
- By development rate: Acute and chronic hydrocephalus.
- By ICP changes: High-pressure, normal-pressure, and low-pressure hydrocephalus (including negative-pressure hydrocephalus).
- By compensatory brain function: Compensatory and decompensated hydrocephalus.
- By pathophysiology: Static and active hydrocephalus.
- By clinical symptoms: Symptomatic and asymptomatic hydrocephalus.
- By location: Extracranial and intracranial hydrocephalus.

From the above, it can be seen that the existing frameworks for the classification of hydrocephalus are mainly based on the causes of cerebrospinal fluid circulation disorders and pressure alterations, e.g., Rekate [3] argued that hydrocephalus is an active distension of the brain ventricular system related to inadequate passage of CSF from its point of production to its point of absorption into the systemic circulation. Building directly on this framework, which emphasizes anatomical flow restriction and models the CSF system as a hydraulic circuit, a classification system is proposed. Kahle et al. [2] discuss the epidemiology, pathophysiology, diagnosis and treatment of hydrocephalus in children, controversies, and the future research agenda for pediatric hydrocephalus. Despite these seminal contributions, prevailing classification systems and clinical guidelines lack explicit criteria for identifying treatment-resistant hydrocephalus.

Numerous studies were reporting relevant cases of RH [11–45], which encompass congenital hydrocephalus, tumor-induced hydrocephalus, infectious hydrocephalus, hemorrhagic hydrocephalus, and other acquired forms, as well as idiopathic hydrocephalus, low-pressure hydrocephalus, and postcranial surgery hydrocephalus. These studies have introduced various novel approaches for managing refractory cases [13, 17, 19, 20, 22, 23, 25, 29–33, 35, 36, 41–43][. However, the definition of RH in the literature remains subjective, often classified as refractory solely based on the challenges associated with clinical treatment. Such a definition is unscientific and fails to support an objective evaluation of standardized diagnostic and therapeutic measures. By reviewing the literature and considering the Diagnosis-Related Group payment system—where treatments exceeding 60 days are automatically grouped for project-based payment—we propose the following definition for RH: progressive hydrocephalus meeting one or more of the following criteria: (1) the absence of significant clinical or radiological improvement within 60 days despite standard interventions, usually due to pathological factors, such as abnormal CSF characteristics; (2) inability to achieve curative surgical treatments attributable to complex anatomy, such as abnormal dynamic changes or multiloculated compartments; and (3) failure to respond after two consecutive therapeutic procedures. RH consists of six distinct subtypes, with infectious hydrocephalus being the most common, followed by low-pressure hydrocephalus. Although there is currently a lack of multicenter data or evidence-based studies supporting this definition of RH, and no uniform definition exists, we anticipate that future application of this criterion will incorporate relevant statistical data and retrospective study results. This will enable the definition to be revised, thereby enhancing its objectivity and applicability.

Our definition of RH differs from the traditional literature’s definition of complex hydrocephalus, though it encompasses the latter’s scope. The traditional definition of complex hydrocephalus often involves abnormal CSF characteristics or circulatory blockages, resulting in excessive accumulation of CSF in two or more separate and non-communicating areas of the ventricular system, accompanied by clinical symptoms [14, 17, 36, 39, 43].

Clarifying the definition of RH will not only ensure recognition of its status as a relatively serious pathological state, which remains challenging to manage across treatment options, thereby promoting clinical attention and the development of appropriate diagnostic and therapeutic strategies, but also provide a basis for prognostic assessment, guide DRG-based payment systems, and facilitate further research on RH.

## Common causes and classification of RH

A key prerequisite for curative surgery in hydrocephalus is the presence of normal CSF characteristics. The primary obstacle to performing curative surgery is CSF infection. Furthermore, curative surgical interventions for hydrocephalus, whether ventriculostomy, lumbar puncture, or ventriculoperitoneal shunting (VPS), typically aim to drain CSF from a single location. When CSF accumulates in multiple regions, as seen in multiloculated hydrocephalus, treatment becomes more complex. Additionally, effective treatment often requires redirecting abnormal CSF accumulation to another location, which depends on the presence of a natural pressure difference. However, the emergence of low-pressure or negative-pressure hydrocephalus has complicated the management of abnormal CSF accumulation, presenting a significant challenge in treatment.

Based on the above considerations and etiology, RH can be classified into the following categories: (1) severe infectious hydrocephalus complicated by ventriculitis [12, 14], with a disease course exceeding 60 days, (2) specific bacterial infectious hydrocephalus [29], (3) complex hydrocephalus [14, 17, 36, 39, 43], (4) low-pressure or even negative pressure hydrocephalus [16, 23, 25, 38], (5) hydrocephalus that fails after two standard surgical treatments, and (6) noninfectious, long-term abnormalities in cerebrospinal fluid properties [16, 21, 28]. The definition in this paper is more therapeutically focused and avoids dividing the complexity of hydrocephalus in terms of infections, occupying lesions, congenital defects, hemorrhage, trauma, or idiopathic causes, as stated by Professor Shizuo Oi, who said that theoretically there are at least 72,576,000 different patterns of hydrocephalus [8]. Also, clinical practice has revealed that RH, as defined in the text, consists of two main types of hydrocephalus: infectious and low-pressure hydrocephalus, so this article focuses on both.

## Core treatment strategy for infectious RH

Infectious RH constitutes a significant proportion of RH cases, and its treatment is central to managing RH overall. The primary challenge in treating infectious RH lies in the contradiction between progressive hydrocephalus and the prolonged inability to improve abnormal CSF characteristics. As such, the treatment strategy primarily focuses on alleviating progressive hydrocephalus and improving CSF characteristics.

To improve CSF characteristics, a comprehensive evaluation of the patient’s condition is essential, including a detailed medical history, CSF analysis, and imaging assessments. Based on this evaluation, pathogen-specific treatment, which aligns with standard antimicrobial therapy, should be initiated. For patients with appropriate conditions, genetic testing of pathogens is highly relevant. CSF analysis should encompass not only routine and biochemical tests but also mandatory cytological examinations [46–48]. Imaging assessments, including contrast-enhanced scans, are critical for detecting abscesses and ventriculitis.

Temporary external drainage is a key method for alleviating the progression of infectious RH. Common temporary external drainage techniques include direct external ventricular drainage (EVD), Ommaya reservoir placement, lumbar cistern drainage, long-tunneled EVD, and modified VPS [30, 32, 42, 49]. Each method offers distinct advantages and disadvantages. Direct EVD is a simple procedure but has limitations, including a short placement duration, uncontrollable drainage volume, a risk of iatrogenic infections, and the inability for the patient to mobilize. Ommaya reservoir placement, while also simple and allowing for a longer placement time, shares similar drawbacks with EVD, including uncontrollable drainage volume, a risk of iatrogenic infections, and immobility. Lumbar cistern drainage is straightforward with a relatively short placement duration, but it similarly presents issues with uncontrollable drainage volume, a risk of iatrogenic infections, and the patient being unable to mobilize. Long-tunneled EVD has a longer placement duration, a lower risk of iatrogenic infections, and a relatively low cost for consumables, but it is still associated with difficult drainage volume control and patient immobility [30, 32, 42]. Modified VPS, though more complex, offers a longer placement duration, controllable drainage volume, and a low risk of iatrogenic infections and allows the patient to mobilize [49]. Among these methods, we recommend the modified VPS [49]. However, it is crucial to comprehensively assess the patient’s condition and economic situation before selecting the most appropriate treatment. Long-tunneled EVD, with relatively low consumable costs, is also a viable option. Currently, we are investigating the combination of the Ommaya reservoir with VPS, in which the peritoneal portion of the shunt is placed externally. Although this procedure is relatively complex, it offers advantages such as a longer placement duration, simultaneous ventricular irrigation, controllable drainage volume, a low risk of iatrogenic infections, and the ability for the patient to mobilize.

## Timing and methods for curative surgery in infectious RH

The timing for curative surgery in infectious RH is determined by the results of CSF sterilization. Since the effectiveness of antimicrobial treatment is solely reflected by the assessment of CSF sterilization, this evaluation must be scientifically rigorous and objectively reliable to guide the decision for curative surgery. Firstly, CSF samples should be collected under conditions that closely mimic the physiological state of CSF circulation. If the CSF is being externally drained, the drainage tube should be clamped for as long as possible (at least 12 h or longer) before collecting the sample, or the sample should be obtained via lumbar puncture. It is crucial to avoid taking the sample directly from the ventricles through the external drainage tube while CSF is actively draining. Secondly, cytological examination of CSF is essential, with particular attention to the presence of plasma cells. Studies have shown that during the progression of bacterial meningitis, a decrease in the total CSF cell count is often accompanied by a reduced proportion of myeloid cells and an increased proportion of lymphocytes, including plasma cells [50]. In our experience, plasma cells can still be detected even when cell counts and biochemical parameters appear normal for a considerable period. Curative surgical treatment performed only after three consecutive CSF tests show no presence of plasma cells has been associated with a very low incidence of reinfection. When feasible, metagenomic next-generation sequencing (mNGS) for pathogen identification in the CSF is a valuable tool [51].

Regarding surgery, VPS is the most commonly used procedure. However, the importance of evaluating the site receiving the cerebrospinal fluid is often overlooked. In fact, the presence of chronic inflammation in the abdomen or a history of conditions that may lead to abdominal adhesions can significantly contribute to the failure of VPS surgery. Therefore, if the abdominal assessment reveals such conditions, they should be considered as contraindications for VPS, and ventriculoatrial shunting should be prioritized instead.

## Difficulties in treating negative pressure or severe low-pressure hydrocephalus

Low-pressure hydrocephalus (LPH) or negative pressure hydrocephalus represents a newly identified variant of hydrocephalus. Patients with this condition exhibit pronounced symptoms of elevated ICP, accompanied by ventricular enlargement. However, measured ICP values are unexpectedly low, typically ≤ 5 cmH_2_O, or even below atmospheric pressure [11, 52–54]. The defining feature of these conditions, however, is the paradox of low ICP coupled with ventricular enlargement. This discrepancy challenges conventional understanding, as the low ICP does not align with the degree of ventricular dilation or the severity of clinical symptoms observed. Diagnosing low-pressure hydrocephalus is often challenging and can initially be misinterpreted as equipment malfunction, leading to multiple unsuccessful interventions such as shunt placement or EVD. Temporary EVD fails to drain CSF when set at standard drainage heights, and shunting is ineffective in transporting CSF from the abnormally low-pressure and dilated ventricles to distal compartments.

All causes of chronic hydrocephalus can be the etiology of LPH, either directly secondary to the primary intracranial disease or slowly developing from VP shunt, so the diagnosis of LPH is often throughout the diagnostic and therapeutic process, and it is very easy to miss or misdiagnose in the early stage. At the same time, its clinical manifestations are often more serious than those before the primary disease or shunt operation, and if it is not treated timely and correctly, it may lead to disastrous prognosis such as long-term bed rest and coma due to the damage of the periventricular structure of the third ventricle.

The mechanisms underlying negative pressure hydrocephalus or severe low-pressure hydrocephalus remain unclear. Several biomechanical hypotheses have been proposed in the literature to explain the LPH phenomenon, such as the viscoelastic principle [53], the “porous sponge model” hypothesis proposed by Hakim et al. [55]. Akins et al. suggested that the changes in the ventricular system in the LPH are essentially analogous to the changes in the negative pressure within the pleural cavity of the lungs during inspiration/expiration [56]. And several scholars have found LPH in patients with cerebrospinal fluid leakage after lumbar puncture in patients with hydrocephalus shunts [57], arachnoid cyst shunts [58], and cerebrospinal fluid leakage after skull base surgery [59], respectively, and have proposed the existence of a pressure gradient difference in the ventricular-subarachnoid space as a reason for the formation of LPH.

Among these,two pathophysiologically relevant hypotheses hold particular clinical significance: decreased cerebral viscoelasticity and increased transcortical pressure gradients [60]. Pang et al. reported recurrence of symptoms in 12 patients with hydrocephalus after cerebrospinal fluid shunt; however, negative pressure extraventricular drainage improved and reversed the symptoms, which led to the theory of altered viscoelasticity [52]. Cheng et al. reported that the elasticity of brain tissue in patients with LPH was significantly lower than that of patients with ordinary hydrocephalus, and that accurate puncture of ventricles intraoperatively became more difficult; postoperative CT examination showed that the brain tissue was unable to regain its elasticity [61]. Suggesting decreased elasticity and increased compliance of brain tissue Olivero et al. Magnetic resonance elastography (MRE) study reported to support this theory [62]. Vassilyadi et al. reported two cases of patients with NePH and proposed the concept of cortical pressure difference [63]. Filippidis et al. analyzed three patients presenting with LPH after skull base surgery, all of whom had cerebrospinal fluid leakage, presumably causing an intercortical pressure difference that triggered LPH[59]. Hamilton and Price, in a study, found by means of an imaging analysis of 20 patients with LPH that cerebrospinal fluid outflow pathways were obstructed in all of them, leading to ventricular and arachnoidal subarachnoid decoupling phenomenon. This decoupling phenomenon further validated the rationality of the theory of cortical pressure difference [64]. However, the above assumptions are still insufficient in guiding clinical practice. For example, based on the hypothesis of “transcortical pressure difference” and a history of lumbar puncture before LPH, lumbar puncture or external lumbar pool drainage is not recommended for patients with LPH, but Lesniak reported that two patients with LPH had good results after lumbar pool abdominal shunt [53], which is obviously contradictory.

The diagnosis of LPH includes the presence of low cranial pressure (≤ 5 cmH_2_O) or even negative pressure by ICP measurement on the basis of the usual presentation of hydrocephalus. If ICP cannot be measured, some imaging features of CT are helpful in the differential diagnosis with normal pressure hydrocephalus. CT imaging shows obvious enlarged ventricles, obvious shrinkage, or disappearance of the subarachnoid space, especially the convexity of the cerebral hemispheres (including the lateral fissure pools), a tension-like spherical sign of the fourth ventricle, the whole brain becomes rigid, the cerebral pools of the annular pools and cranio-cervical junction area are significantly shrunken or blurred or disappeared, and the conventional cerebrospinal fluid shunt is ineffective; LPH should be considered. Ineffective, LPH should be considered and requires further validation. A history of traumatic brain injury (TBI) or subarachnoid hemorrhage, especially with central nervous system (CNS) infections, should be of particular concern for the presence of LPH [65].

The emergence of low-pressure hydrocephalus and negative pressure hydrocephalus presents a new challenge in determining the optimal destination for diverting the abnormal accumulation of CSF. Currently, the peritoneal cavity is the most common site for CSF drainage. However, intra-abdominal pressure typically ranges from 0 cmH_2_O (atmospheric venous pressure) to 8 cmH_2_O under normal clinical conditions (1 mmHg = 1.33 cmH_2_O). In contrast, draining CSF into the atrium exposes it to central venous pressures ranging from 5 to 12 cmH_2_O. In patients with LPH or NegPH, cerebral compliance and ventricular pressure are first improved, and ventricular size and morphology are restored as much as possible by precise external CSF drainage [7]. Significant variation exists in the initial external ventricular drain height setting, ranging from − 20 to − 5 cmH_2_O, achieved by one or more large-volume ventricular cerebrospinal fluid aspirations (30–50 ml), followed by drainage of cerebrospinal fluid at a fixed volume of 10–15 ml per hour. After the patient is clinically stable and the ventricles have shrunk in size, a CT or MRI (e.g., rapid sequence MRI) scan of the head is performed to document the baseline ventricular size. The cerebral compliance is adjusted by gradually increasing the drainage strength (high) (1–2 cm every 3–5 days), and a stepwise decision is made when the EVD drainage height reaches above the level of the external auditory canal (≥ 0 cmH_2_O, with some authors suggesting 4 cmH_2_O [7]), and clinical improvement is stabilized. Discontinuation and removal of the EVD will require further permanent shunt therapy in most patients, and a minority of obstructive hydrocephalus and non-inflammatory hydrocephalus may still be amenable to ETV therapy [6]. The choice of a pressurized shunt tube or low-pressure tube for permanent shunting has also been reported as a successful choice of valveless shunt treatment in specific cases [9], although overshunting has not been found in the reports, and the author’s practice has not identified this as a problem. However, the problem of excessive shunting should still be highly concerning in valve-free shunting. Meanwhile, it has been pointed out that a triple ventriculostomy is feasible to balance the ventricular-subarachnoid pressure gradient for adjuvant treatment of low cranial pressure hydrocephalus[9].

## Future strategies for treating RH

Although progress has been made in the treatment of RH, significant challenges remain, posing a serious threat to the survival of hydrocephalus patients and representing the primary cause of treatment failure. Therefore, RH requires substantial attention.

Firstly, as infection is the leading cause of RH, with many cases involving reinfection after shunting, rapid pathogen evaluation and drug resistance analysis are fundamental to successful treatment. Secondly, selecting the appropriate temporary external drainage method is crucial for optimizing the conditions for subsequent treatment. Thirdly, effective, standardized, and comprehensive treatment strategies, including adjunctive therapies and rehabilitation, should be explored. These approaches should aim to achieve CSF sterilization within a short time and create favorable conditions for curative treatment. Fourthly, objective indicators to assess CSF sterilization results need to be identified. Fifthly, clear criteria and methods for performing curative treatments for RH should be established.

Additionally, low-pressure hydrocephalus and negative pressure hydrocephalus, as unique subtypes of hydrocephalus, have mechanisms that remain unclear. Further research should focus on understanding their underlying causes, particularly CSF hydrodynamics. The primary goal should be to restore the elasticity of brain tissue through temporary external drainage. Proper timing of shunting is critical, requiring the establishment of a pressure gradient between the ventricles and the site for receiving the abnormally accumulated CSF. The development of specialized shunting devices is essential to effectively address these unique challenges.

Short-term research priorities will optimize RH management through improving the efficiency of anti-infective treatment, establishing a rapid pathogen detection and drug resistance analysis process, clarifying the cure of the infection by combining the CSF and imaging objective indexes, and standardizing the treatment protocols to improve the success rate of RH cure. Future long-term research involves basic research on the pathogenesis of different RH, technological breakthroughs in the development of special subtypes of triage devices, and drug development.

## Conclusion

RH exists across different types of hydrocephalus and is clinically recognized as a distinct form of hydrocephalus. According to the Diagnosis-Related Group payment system, RH is defined as progressive hydrocephalus that cannot be improved within 60 days due to pathological factors, such as abnormal CSF characteristics, and cannot be treated with curative surgical procedures, progressive hydrocephalus due to multiloculated hydrocephalus or abnormal CSF dynamics that cannot be effectively managed with simple shunting or endoscopic ventriculostomy, or hydrocephalus that fails after two treatment attempts. RH includes six subtypes, with infection being the most common cause, followed by low-pressure hydrocephalus. Early subtype identification may reduce futile interventions.

**Fig. 1:**
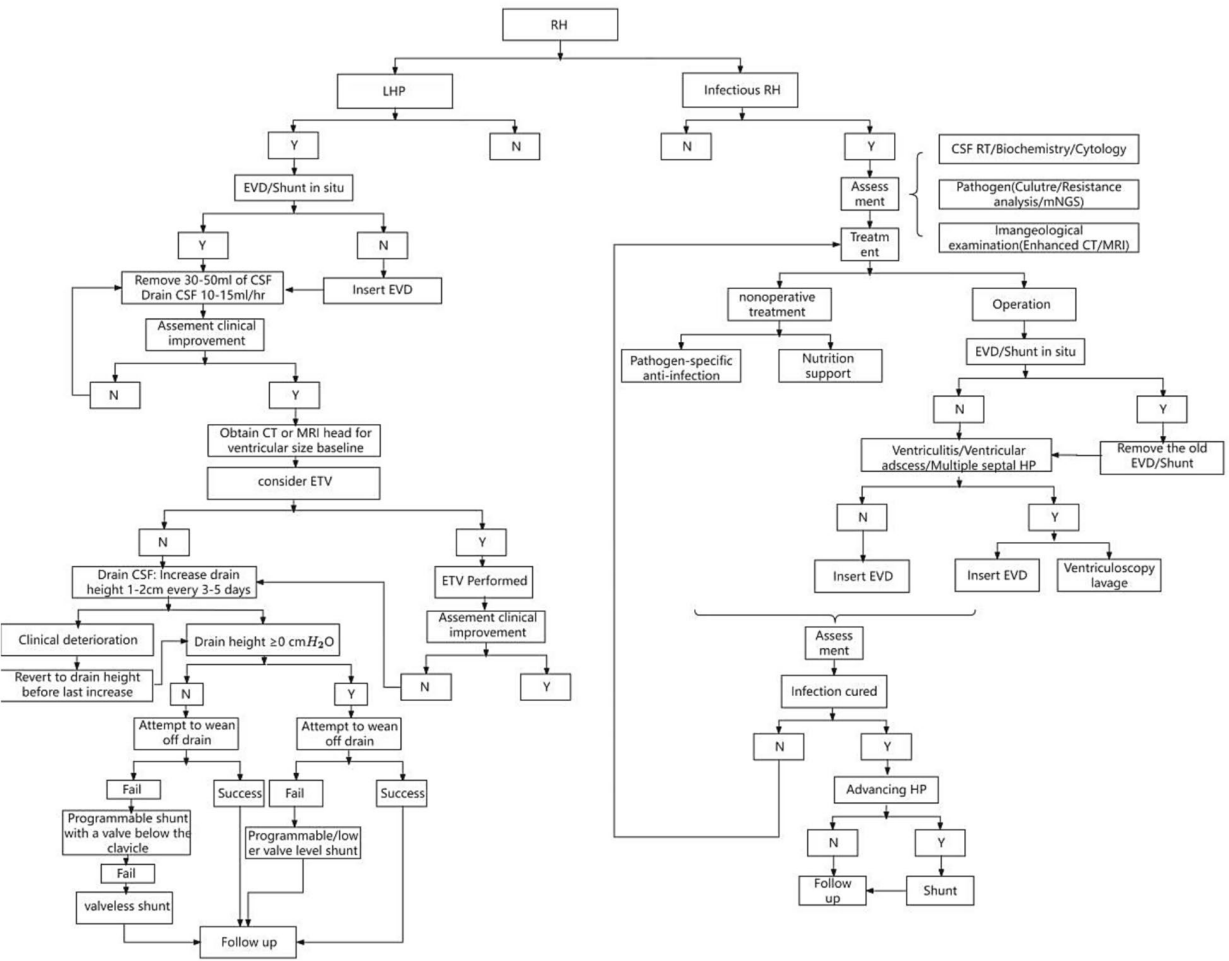
Flowchart for diagnosis and treatment of infectious/low-pressure RH

When selecting temporary measures for addressing progressive hydrocephalus, it is essential to consider both the strengths and weaknesses of the available methods, as well as the patient characteristics. In infectious RH, CSF sterilization and the assessment of the results are critical. Curative surgery for infectious RH should only be performed after CSF has been completely sterilized to normal levels. In the diagnosis and treatment of low-pressure RH, the key challenge is determining the appropriate site for receiving the CSF. The diagnostic flowchart is shown in Fig. 1.

## Data Availability

Not applicable.

## Abbreviations

CSF: Cerebrospinal fluid
ICP: Intracranial pressure
VPS: Ventriculoperitoneal shunting
Mngs: Metagenomic next-generation sequencing
LPH: Low-pressure hydrocephalus
TBI: Traumatic brain injury
RH: Refractory hydrocephalus
EVD: External ventricular drainage
MRE: Magnetic resonance elastography
